# Characterization of plasma metabolites and proteins in patients with herpetic neuralgia and development of machine learning predictive models based on metabolomic profiling

**DOI:** 10.3389/fnmol.2022.1009677

**Published:** 2022-10-06

**Authors:** Ruihao Zhou, Jun Li, Yujun Zhang, Hong Xiao, Yunxia Zuo, Ling Ye

**Affiliations:** ^1^Department of Pain Management and National Clinical Research Center for Geriatrics, West China Hospital, Sichuan University, Chengdu, China; ^2^Department of Anesthesiology, West China Hospital, Sichuan University, Chengdu, China

**Keywords:** herpes zoster, postherpetic neuralgia, herpetic neuralgia, proteomics, metabolomics, machine learning, predictive model

## Abstract

Herpes zoster (HZ) is a localized, painful cutaneous eruption that occurs upon reactivation of the herpes virus. Postherpetic neuralgia (PHN) is the most common chronic complication of HZ. In this study, we examined the metabolomic and proteomic signatures of disease progression in patients with HZ and PHN. We identified differentially expressed metabolites (DEMs), differentially expressed proteins (DEPs), and key signaling pathways that transition from healthy volunteers to the acute or/and chronic phases of herpetic neuralgia. Moreover, some specific metabolites correlated with pain scores, disease duration, age, and pain in sex dimorphism. In addition, we developed and validated three optimal predictive models (AUC > 0.9) for classifying HZ and PHN from healthy individuals based on metabolic patterns and machine learning. These findings may reveal the overall metabolomics and proteomics landscapes and proposed the optimal machine learning predictive models, which provide insights into the mechanisms of HZ and PHN.

## Introduction

Herpes zoster (HZ) is caused by reactivation of the herpes virus, which occurs during aging or immunosuppression and presents as a localized, painful cutaneous eruption (Sampathkumar et al., 2009; Bader, 2013). The annual incidence of HZ is 2–5/1,000 people per year (Johnson and Rice, 2014). Furthermore, cancer and immune dysfunction may increase the incidence of HZ (Johnson and Rice, 2014). Postherpetic neuralgia (PHN) is the most common chronic complication of HZ, with an incidence rate of up to 30%, and is defined as pain lasting more than 3 months after the rash has healed (Sampathkumar et al., 2009; Johnson and Rice, 2014; Yang et al., 2019). However, the incidence of PHN in patients with HZ over 50 years of age is still as high as 25–30% (Sampathkumar et al., 2009). Once PHN develops, treatment is extremely difficult and less effective, and patients suffer from severe physical and psychological disabilities due to continuous pain (Dworkin et al., 2010). Considering the tremendous financial responsibility on patients, society, and the medical system of PHN, exploring the risk factors and elucidating the mechanisms associated with the early diagnosis, classification of risks, and prediction of outcomes between HZ and PHN underlying their clinical development is urgently required.

Currently, we lack precise indicators for screening high-risk PHN populations, and early implementation of preventive interventions is impossible (Delaney et al., 2009). The main reason is that the pathogenesis of PHN is still not comprehensive and thorough, and the neuropathy caused by viral infection has its specificity (Delaney et al., 2009). Researchers explored the factors related to PHN based on extensive data analysis and built a prediction model in hospitalized patients with HZ, identifying 62 variables related to PHN. These models exhibited high accuracy values (Li et al., 2020). The development of omics technology based on large-sample sequencing has provided a potential new method for predicting PHN (Freidin et al., 2016; Wang T. et al., 2020). Our previous results revealed that we should focus on the outcome of HZ and the prediction and intervention of PHN from the metabolism and immunity perspective because the susceptibility of PHN in the elderly is related to metabolic and immune dysfunction. Metabolomics and proteomics, as components of systems biology, can be used to explore the molecular mechanism and from the immune-metabolism perspective of pain, is an effective research method to identify biomarkers and provide new insights into the generation and development of PHN (Shen et al., 2020; Jiang et al., 2021).

**FIGURE 1:**
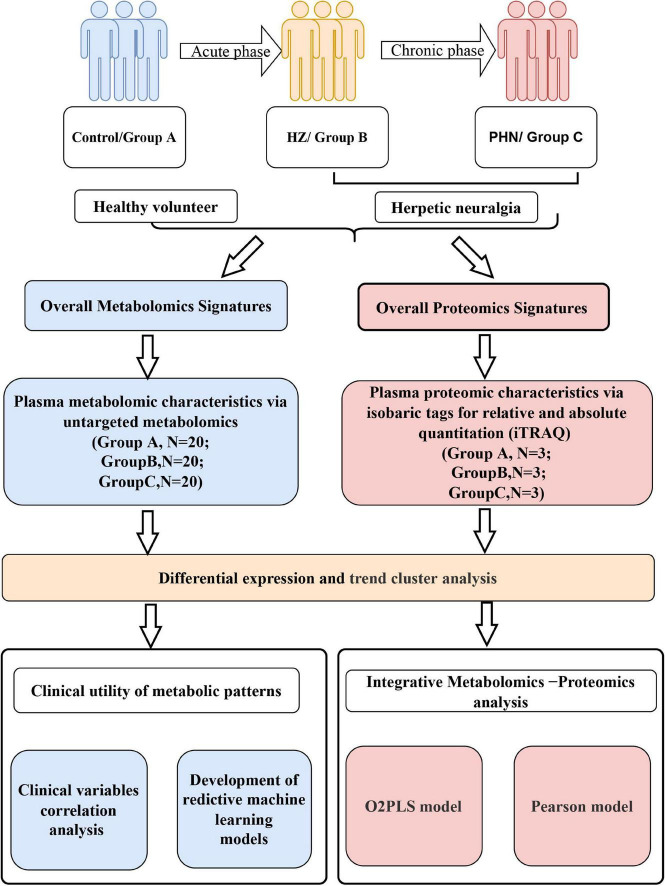
Flowchart depicting metabolomic and proteomic characteristics of herpes zoster and postherpetic neuralgia.

In this study, we applied untargeted metabolomics and quantitative proteomics using isobaric tags for relative and absolute quantitation (iTRAQ) of plasma from healthy, HZ, and PHN participants (Figure 1). In addition, we identified differentially expressed metabolites (DEMs), differentially expressed proteins (DEPs), and key signaling pathways that differed between healthy volunteers and individuals with acute and/or chronic herpetic neuralgia.

Furthermore, we developed and validated three optimal predictive models based on metabolic patterns and machine learning. We aimed to describe the metabolomic and proteomic signatures of herpes virus infection-induced neuropathy. Additionally, our study can help determine the individual differences that influence pain symptoms and drug reactions in HZ and PHN.

## Materials and methods

### Study population and setting

Patients with HZ and PHN were obtained from the pain management department of the West China Hospital of Sichuan University. The study was conducted according to the Code of Ethics of the World Medical Association (Declaration of Helsinki). All patients signed an informed consent form, and the Ethics committee of West China Hospital of Sichuan University approved the implementation of the study, which was registered at the China Clinical Trials Registry (Registration No. ChiCTR1800015561). The patient plasma samples were collected between September 2017 and September 2018. The West China Hospital of Sichuan University selected 60 participants: 20 HZ patients, 20 PHN patients, and 20 healthy individuals.

The inclusion criteria of HZ and PHN cohort were as following: (1) Over 18 years old; (2) Diagnosed with HZ or PHN; (3) Visual analogue scale (VAS) > 3 points before treatment; (4) Accompanied by neuropathic pain in the lesion area; (5) Without serious complication. The exclusion criteria of HZ and PHN cohort were as following: (1) Multiple segments involved; (2) With severe cardiovascular disease, pulmonary disease or abnormal liver and kidney function; (3) Severe pain caused by other diseases; (4) Alcohol and drug abusers; (5) With mental disorders and cannot obtain informed consent.

The healthy control group was recruited from healthy people and had no blood relationship with the patient group. The healthy control group was matched with the patient group according to age and gender. At the same time, the following criteria should be met: (1) No immunosuppressive agents, hormones or biological agents were used in the last month; (2) No history of cancer, AIDS and other diseases; (3) Patients with severe heart, liver and kidney failure and severe infection were excluded.

### Untargeted metabolomics analysis

The samples were divided into three groups: group A (healthy control), group B (HZ), and group C (PHN), and each group contained 20 samples. Metabolomic analysis was based on previously reported methods (Jiang et al., 2019). In addition, liquid chromatography-mass spectrometry (LC-MS) analyses were performed using a UHPLC system (1290, Agilent Technologies), and the specific parameters for the positive ion mode (POS) and negative ion mode (NEG) are shown in Supplementary Methods. Statistical analysis and data presentation mainly included normalizing the original data, identifying metabolites *via* database and multivariate statistical analysis [principal component analysis (PCA), partial least squares discriminant analysis (PLS-DA), and orthogonal projection to latent structures-discriminant analysis (OPLS-DA)].

The variable importance in the projection (VIP) of PLS-DA model variables and the *p*-value for the univariate statistical analysis *t*-test were applied to screen significant DEMs among different comparison groups (VIP ≥ 1 and *t*-test *P* < 0.05) (Ivanisevic and Want, 2019). In addition, we performed a clinical variable correlation analysis of differential metabolites using the Pearson correlation coefficient, including the basic information of patients such as sex, age, course of the disease, site of disease, and complications, and the VAS score.

### Proteomics analysis

We selected three representative samples from each of the three previously collected populations (Group A, B, C) for proteomic determination, and each group had three samples: A1, A2, A3, B1, B2, B3, C1, C2, and C3. The details of protein digestion, iTRAQ labeling, and analysis are provided in Supplementary Methods, and we have referred to previous studies (Ren et al., 2017; Zhao et al., 2018). Briefly, it includes protein digestion, iTRAQ labeling peptide, High PH Reverse Phase separation, low PH nano-HPLC-MS/MS analysis, and identification and quantification of peptides and proteins using the Mascot Distiller (Version 2.6) and Scaffold (Version 4.7.2) databases. Proteins that changed by > 1.2 or < 0.83 and *p* < 0.05 were considered DEPs.

### Predictive model construction

We used the metabolite level as a candidate feature and applied the least absolute shrinkage and selection operator (LASSO) (Tibshirani, 1996) *via* the “glmnet” R package to select the feature in the training data. The penalty parameter λ of the model is determined by calculating the 10-fold cross-validation and selecting the one with the smallest partial likelihood deviation λ-value (lambda. min) for metabolite screening to obtain the metabolites used for modeling.

Subsequently, three mature machine learning algorithms (Yuan et al., 2012; Han et al., 2014), random forest (RF) (Breiman, 2001), support vector machine (SVM) (Noble, 2006) and logistic regression (LR), were used to predict groups A, B, and C, respectively (each group was compared as a binary outcome). Five-fold cross-validation was applied to the modeling process of the three machine-learning methods. In addition, we plotted the ROC curve using the pROC package (Robin et al., 2011), in which the AUC value was used to evaluate the effectiveness and predictive power of the model.

### Short time-series expression miner analysis

Trend analysis is a cluster analysis method used to explore the expression patterns of differential metabolites and proteins in multiple samples with continuous characteristics. As described earlier, the three groups, A, B, and C, could be considered the normal, acute, and chronic phases of the disease progression process. We applied the STEM clustering algorithm (Ernst and Bar-Joseph, 2006) to identify metabolites/proteins that match certain biological characteristics and then generalized and classified the expression trends of DEMs/DEPs according to the analysis results.

### Integrative proteomics-metabolomics analysis

To obtain the significant proteins and metabolites that affect the sample grouping and analyze the correlation characteristics, two models, including the O2PLS (bidirectional orthogonal projections to latent structures) (Bouhaddani et al., 2016) and Pearson models (details shown in Supplementary Methods), were further applied to analyze protein expression and metabolite abundance.

## Results

### Basic characteristics of study cohorts

We collected the demographic data of all the individuals, including 20 healthy volunteers, 20 patients with HZ, and 20 patients with PHN. The demographic data of the three groups were collected, including sex, age, degree of pain, skin lesion location, disease course, and disease history (Table 1). No significant differences were observed in age, sex, disease history, or other general characteristics among the three groups. The disease course in the HZ group was less than 3 months, consistent with the definition of HZ. Regarding skin lesions, lesions in the trunk (thoracic nerve region) were the most common in the HZ (*n* = 10, 50%) and PHN groups (*n* = 13, 65%).

**TABLE 1:** Characteristics of the study population.

	Control (*n* = 20)	HZ (*n* = 20)	PHN (*n* = 20)
Age, mean ± SD (y)	64.22 ± 11.56	66.10 ± 13.49	69.55 ± 11.15
**Sex, n (%)**			
Male	10 (50%)	11 (55%)	10 (50%)
Female	10 (50%)	9 (45%)	10 (50%)
**Disease Course (M)**			
<3 M	0	20	
4–6 M	0	0	10 (50%)
7–12 M	0	0	3 (15%)
>12 M	0	0	7 (35%)
**Location, n (%)**			
Face (Trigeminal nerve region)	0	4 (20%)	1 (5%)
Neck and upper limbs (Cervical nerve region)	0	1 (5%)	4 (20%)
Trunk (Thoracic nerve region)	0	10 (50%)	13 (65%)
Buttocks and lower limbs (Lumbar and Sacral nerves region)	0	5 (25%)	2 (10%)
**Disease history**			
Hypertension	1 (5%)	2 (10%)	3 (15%)
Diabetes	0	1 (5%)	3 (15%)
Immune related diseases	0	0	3 (15%)
Osteoporosis	0	0	0
COPD	0	2 (10%)	1 (5%)

### Overall metabolomics signatures of disease progression in patients with herpes zoster and postherpetic neuralgia

A total of 2,280 metabolites were identified in POS and NEG ionization modes during metabolite detection. The overlapping display analysis of the base peak chromatogram (BPC) of different quality control (QC) and blank samples suggests that the detection instrument is stable, and no cross-contamination was observed between samples. In addition, all QC samples were within ± 2 times the standard deviation (Supplementary Figures 1A,B), and no significant peak was detected in the blank samples (Supplementary Figures 1C,D).

As previously mentioned, multivariate statistical analyses of these metabolites were performed using PCA, PLS-DA, and OPLS-DA. First, the PCA score chart for the POS and NEG modes (Supplementary Figure 2) exhibited evident metabolic differences between the control and disease groups. In addition, PLS-DA was used to maximize the distinction between groups and detect the model’s fitness. The three groups were also well separated under supervised conditions in both modes (Supplementary Figure 3). To avoid over-fitting in multivariate statistical analysis, we performed OPLS-DA and further validated the results using a permutation test. The permutation test revealed that the original Q2 value among the three groups was mostly lower than the Q2 value after model replacement (Supplementary Figure 4). Collectively, these analyses indicated that the model was reliable and did not overlift.

**FIGURE 2:**
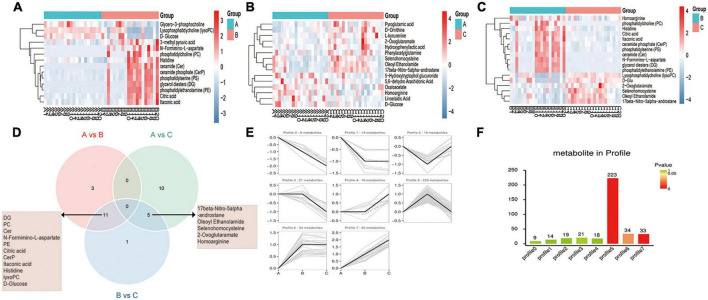
Metabolic patterns in healthy control, HZ, and PHZ groups. **(A–C)** The heatmap of differential expressed metabolites (DEMs) between groups: **(A)** Groups A vs. B, identifying 14 DEMs, **(B)** group A vs. C, identifying 15 DEMs, and **(C)** groups B vs. C, identifying 17 DEMs. Red represents increased expression; blue represents decreased expression. **(D)** Identification of significant common metabolites *via* Venn diagram. **(E)** The trend expression profile of metabolites in the three groups. The black line represents the trend line, and the gray line represents the expression trend of each metabolite. **(F)** Number of metabolites in the profile. The height of the column represents the number of metabolites, and the color of the column represents the *P*-value.

DEMs were screened by pairwise comparison between the three groups and are depicted as heatmaps (Figures 2A–C). We also identified common metabolites among the three groups using a Venn diagram (Figure 2D). Notably, 11 metabolites were identified at the intersection of groups A vs. B and B vs. C, which might be more relevant to acute or acute and chronic pain transformation. In addition, five metabolites were identified at the intersection of groups A vs. C and B vs. C, which might be more relevant to chronic or acute, and chronic pain transformation. We further conducted a KEGG metabolite pathway enrichment analysis to explore the main biochemical metabolism and signaling pathways involved in the DEMs between the groups (Supplementary Table 1). Notably, retrograde endocannabinoid signaling, glycerophospholipid metabolism, and arachidonic acid metabolism were enriched in groups A vs. B and B vs. C, which might play a vital role in the progression of the disease. In addition, we clustered the metabolic expression patterns by STEM analysis and obtained three significant metabolite expression profiles (Figures 2E,F, profiles 5, 6, and 7). Furthermore, the results suggested that metabolites of profile 5 (unchanged in the healthy and PHN groups and increased in the HZ group) were mainly involved in lipid metabolism, tricarboxylic acid cycle, and amino acid metabolism (Supplementary Table 2).

**FIGURE 3:**
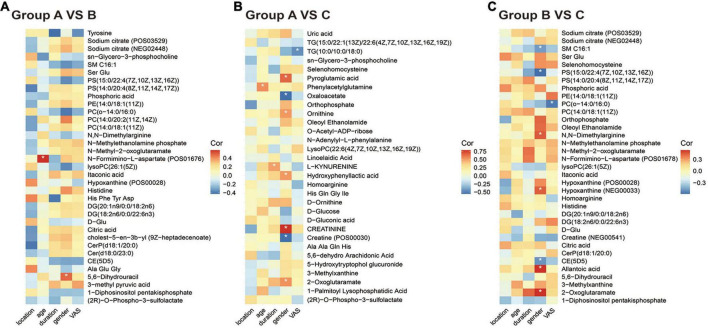
Correlation heatmap between metabolic patterns and clinical data. **(A–C)** The heatmap of altered metabolic patterns between groups and clinical data: **(A)** Groups A vs. B, **(B)** groups A vs. C, **(C)** groups B vs. C. Clinical data including VAS, age, sex, location, and duration.

Finally, we analyzed the correlation between differential metabolites and clinical data. As shown in Figure 3A, the expression of N-formimino-L-aspartate increased with age (*r* = 0.5873, *P* = 0.0064), and 5,6-Dihydrouracil was increased in women (*r* = 0.488, *P* = 0.028) in group B vs. A. In the PHN group (Figures 3B,C), nine metabolites were increased in women and creatinine registered the highest correlation (*r* = 0.790, *P* < 0.05), whereas five metabolites were increased in men and phosphatidylserine (PS) exhibited strong relevance (*r* =−0.585, *P* < 0.05). L-kynurenine expression was positively correlated with disease duration (*r* = 0.500, *P* < 0.05). The expression of phenylacetylglutamine was positively correlated with age (*r* = 0.548, *P* < 0.05). Interestingly, the expression of triglycerides (TG) and phosphatidylcholine (PC) was negatively correlated with the degree of pain (*r* =−0.453, −0.552, *P* < 0.05).

### Development of the Predictive model of herpes zoster and postherpetic neuralgia based on metabolic patterns and machine learning

**FIGURE 4:**
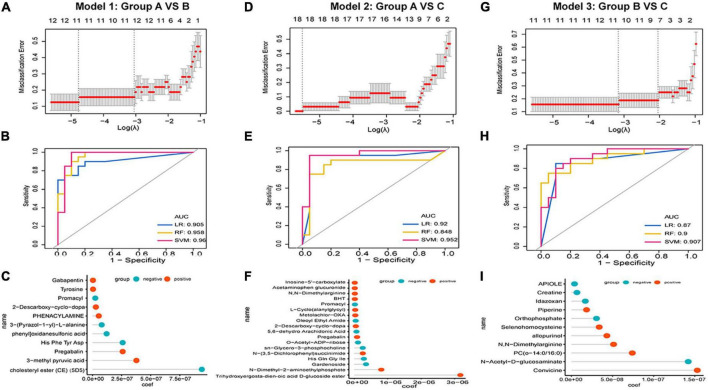
Development of predictive models in HZ and PHN based on metabolic patterns and machine learning. **(A–C)** Development of predictive model 1 group A vs. B: **(A)** LASSO regression, **(B)** the receiver operating characteristic curves of three classifiers, **(C)** correlation coefficient of specific metabolite in the model; **(D–F)** development of predictive model 1 Model 2 group A vs. C: **(D)** LASSO regression, **(E)** the receiver operating characteristic curves of three classifiers, **(F)** correlation coefficient of specific metabolite in the model; **(G–I)** development of predictive model 1 Model 2 group A vs. C: **(G)** LASSO regression, **(H)** the receiver operating characteristic curves of three classifiers, **(I)** correlation coefficient of specific metabolite in the model.

We applied a meticulous machine-learning approach to assess the clinical utility of differentiating metabolic patterns in HZ/PHN to assess the predictive power of specific expressed metabolic patterns in classifying from healthy to HZ and PHN. For groups A vs. B, we performed LASSO logistic analysis to select features in the training dataset and established an 11-metabolite predictive model to classify healthy controls and HZ patients (Figure 4A). Strikingly, we observed that the specific metabolic profile could accurately classify healthy controls and HZ patients in model 1 (LR, AUC = 0.905, RF, AUC = 0.958, SVM, AUC = 0.96 (Figure 4B). Among the 11 features in the diagnostic signature, cholesteryl ester (CE) demonstrated the highest correlation coefficient for healthy people in predicting HZ (Figure 4C). For group A vs. C (Figures 4D–F), an 18-metabolite predictive model (model 2) was used to classify healthy controls and PHN patients (LR, AUC = 0.92, RF, AUC score = 0.848, SVM, AUC = 0.952). For group B vs. C (Figures 4G–I), the predictive model identified 11 metabolites (LR, AUC = 0.87; RF, AUC = 0.9; SVM, AUC = 0.907). Furthermore, convicine registered the highest correlation coefficient with HZ in predicting PHN.

### Proteomic Signatures for disease progression in patients with herpes zoster and postherpetic neuralgia

**FIGURE 5:**
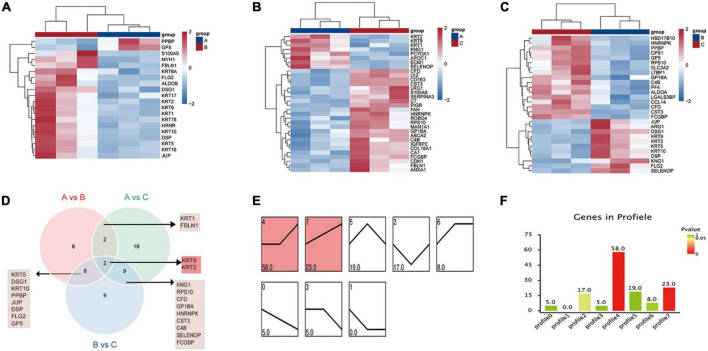
Protein expression patterns in healthy control, HZ, and PHZ groups. **(A–C)** The heatmap of differential expressed proteins (DEPs) between groups: **(A)** Group A vs. B, **(B)** group A vs. C, **(C)** group B vs. C. **(D)** Identification of significant common proteins *via* Venn diagram; **(E)** trend expression profile of proteins among three groups; **(F)** number of proteins in the profile. The height of the column represents the number of proteins, and the color of the column represents the *P*-value.

DEPs were screened by pairwise comparison between the three groups and are depicted as heatmaps (Figures 5A–C). In addition, we identified common DEPs among the three groups using a Venn diagram (Figure 5D). Notably, keratin 2 (KRT2) and KRT9 were identified at the intersection of three gene lists, which were subgroups of the keratin family related to cell structure and integrity and might be relevant to the disease progression of viral infection-induced neuropathy. Furthermore, we performed a KEGG enrichment analysis of the DEPs between the groups (Supplementary Figures 5A–C). In groups A vs. B, DEPs were mainly enriched in ECM-receptor interactions, viral protein interactions with cytokines and cytokine receptors, and chemokine signaling pathways. In groups A vs. C, DEPs were mainly enriched in critical pathways, such as complement and coagulation cascades, cholesterol metabolism, and neutrophil extracellular trap formation. The Gene Ontology (GO) term enrichment analysis results among groups are shown in Supplementary Figures 5D–F. In groups A vs. B, skin development, epidermal development, keratinization, and neutrophil-mediated immunity were mainly enriched, consistent with the clinical features of HZ with painful herpes and immunosuppression. Neutrophil-mediated immunity-related biological processes play vital roles in PHN.

Finally, we performed STEM analysis and obtained two significant expression profiles (Figures 5E,F, profiles 4 and 7). Bioinformatics analysis of the proteins assigned to profile 4 (unchanged in the control and HZ and increased in the PHN) was mainly related to viral infection and immune reaction (Supplementary Table 3). Bioinformatic analysis of the proteins assigned to profile 7 (continuous increase in expression level) was mainly related to inflammatory chemotaxis and neuroimmune inflammation (Supplementary Table 4).

### Integrative metabolomics–proteomics analysis in healthy control, herpes zoster, and postherpetic neuralgia groups

To better characterize the multi-omics of HZ and PHN during disease progression, we performed model correlation analysis based on protein expression and metabolite abundance.

**FIGURE 6:**
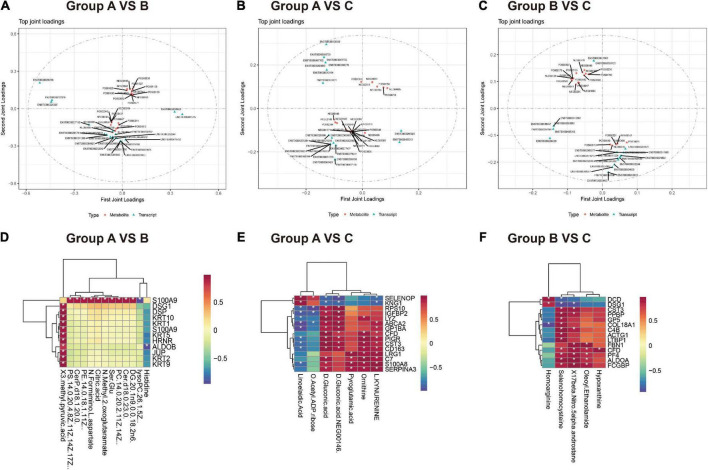
Integrative metabolomics-proteomics analysis in healthy control, HZ, and PHZ groups. **(A–C)** Loadings plots of differentially expressed metabolites and proteins altered in differential compared groups. **(A)** Group A vs. B, **(B)** group A vs. C, **(C)** group B vs. C. The loading value represents the explanatory ability of the variable (metabolite/protein) in each component, and the positive and negative values represent a positive or negative correlation with other omics. The greater the absolute value of the load value, the stronger the correlation. **(D–E)** Correlation heatmap between differential metabolites and proteins among groups. **(D)** Group A vs. B, **(E)** group A vs. C, **(F)** group B vs. C. Red represents increased expression, and blue represents decreased expression.

(1) O2PLS model: Based on the element loading value (loading value represents the correlation of omics data) results, we filtered the proteins and metabolites with the top 25 loading values squared in the first two dimensions to integrate the loading diagram and identify the proteins and metabolites with the greatest degree of association (Figures 6A–C). In groups A vs. B (Supplementary Table 5), the metabolites with strong correlations were mainly lipid metabolites (CerP, PC, PS, and PE). The proteins with strong correlations were mainly various keratin components and cytokines. In groups A vs. C (Supplementary Table 6), metabolites with strong associations were mainly fatty acid (5,6-dehydro arachidonic acid and linoleic acid) and amino acid metabolites (ornithine, pyroglutamic acid, and l-kynurenine). Proteins with strong associations were mostly associated with neuroimmunity and inflammation (LRG1, S100A9, FAH, SERPINA3, C7). In addition, the TOP 5 metabolites (histidine, selenocysteine, glycerol phosphatidylcholine, PS, and PC) and TOP 5 proteins (ARG1, PF4, GP1BA, SELENOP, and C4B) were identified in groups B and C (Supplementary Table 7).

(2) Correlation coefficient model: Correlation heatmaps between differential metabolites and proteins among groups are shown in Figures 6D–F. For example, in groups A and HZ (Figure 6D), some noteworthy issues were identified: (1) S100A9 was highly positively correlated with the expression of lipid metabolites, such as DG, Cer, PS, PE, and PC. (2) Cytoarchitectural proteins, such as keratin components, JUP, and DSG1, were positively correlated with 3-methyl pyruvate.

In groups A and PHN (Figure 6E): (1), some proteins associated with immune inflammation, such as CST3, CD163, and S100A9, were highly positively correlated with the metabolite d-ornithine (cor > 0.95). (2) Inflammatory mediator proteins, such as S100A8 and SERPINA3, were positively correlated with L-kynurenine. Comparing the PHN group with the HZ group (Figure 6F), we observed that proteins and metabolites strongly correlate, including inflammation-related proteins such as C4B, CST3, GP5, PPBP, and LTBP1, were positively correlated with selenium homocysteine expression.

## Discussion

To the best of our knowledge, this is the first attempt to discover metabolomic and proteomic signatures of disease progression in patients with HZ and PHN. We identified DEMs, DEPs, and signaling pathways that transition from healthy volunteers to various stages of HZ and PHN, similar to the acute and chronic phases of herpetic neuralgia. Moreover, based on metabolic patterns and machine learning, we developed and validated three optimal predictive models for herpes virus infection-induced neuropathy. Furthermore, we performed integrative metabolomics-proteomics analysis to explore the molecular mechanism from the protein-metabolism perspective, which provides new insight into the generation and development of PHN.

In this study, alterations in plasma metabolites and proteins may distinguish patients with HZ from controls. Regarding metabolomics, the dysregulated metabolites were mostly related to these four metabolic pathways: lipid metabolism, fatty acid metabolism, histidine metabolism, and the tricarboxylic acid cycle. The upregulated lipid metabolites are commonly upregulated in neuroinflammatory responses and are vital regulators of pain perception (Malan and Porreca, 2005; Neely et al., 2012). In particular, the Cer-S1P pathway has emerged as a vital modulator and druggable target for developing peripheral and central sensitization involved in pain processing (Salvemini et al., 2013). Meanwhile, the downregulated lipid metabolite (lysoPC) was identified as a metabolic biomarker for multisite musculoskeletal pain (MSMP) by metabolomics (Liu et al., 2021). Furthermore, our results suggest that increased levels of citric acid and pyruvate, and decreased D-glucose, might indicate that inflammatory stimuli could lead to increased glucose uptake by peripheral tissues and disrupt the tricarboxylic acid cycle, affecting the energy metabolism of the body.

Regarding proteomics, the dysregulated proteins in patients with HZ were mostly highly sensitive markers of the innate immune system or inflammation-related diseases. Notably, S100A9 was overexpressed in the dorsal root ganglia (DRGs) after HSV-1 infection *via* the TLR4/TNF pathway and positively modulated acute herpetic neuralgia, consistent with our results (Silva et al., 2020). Integrative analysis revealed that S100A9 expression was positively correlated with lipid and tricarboxylic acid cycle-related metabolites. S100A9 may also exert its biological effects through the phospholipid transmembrane and participate in the acute inflammatory response process *via* the lipid metabolic pathway with enhanced energy metabolism. Meanwhile, many upregulated proteins related to cell structure and integrity and epithelial tissue damage (DSP, JUP, DSG1, and KRT family) were also detected in HZ patients, which resulted in the secretion of large amounts of keratin and connexin components by proliferating or apoptotic epithelial cells and blister formation on the body surface (Jones et al., 2014; Tommasi et al., 2020). In summary, metabolomics indicated that increased lipid metabolism and abnormal energy metabolism due to viral infection might be the main manifestations of HZ. Alterations in proteomics indicated that virus infection contributed to the immune-inflammatory response in epithelial tissues and DRGs, and secretion of keratin and connexin components released large amounts of inflammatory factors to promote the inflammatory response further.

Regarding metabolomics in PHN patients, the dysregulated metabolites were mostly related to four metabolic pathways: fatty acid, tryptophan, glutamate, and ornithine. Dysregulated fatty acid metabolites were identified as vital inflammatory mediators of arachidonic acid, and its metabolites could activate the endogenous transmitter TRPV1 to increase the sensitivity of nerves (Zimmer et al., 2018; Kim et al., 2019; Osthues and Sisignano, 2019). More importantly, the activation of the tryptophan metabolic pathway by inflammatory stimuli in the PHN patients led to abnormalities in 5-HT metabolism and reuptake and might respond to the absence of the action of the downstream inhibitory system and increased neuro sensitivity leading to the development of PHN (Roth et al., 2021). Consistent with this, some DEMs were identified to be involved in the metabolic processes of essential neurotransmitters *in vivo*, including glutamate, glutamine, and aspartate, which might lead to abnormal synthesis and release of the excitatory neurotransmitter glutamate, and thus be involved in the development of neurosensitization (Hoffmann and Charles, 2018).

Regarding proteomics in patients with PHN, some upregulated proteins (HNRNPK and RPS10) were related to viral infection and replication (Pettit Kneller et al., 2009; Sundaramoorthy et al., 2021). This implies that viral infection might remain a cause of changes in the disease during the PHN phase, and studies have proven that higher viral DNA loads contribute to the risk factors for PHN (Quan et al., 2006; Quinlivan et al., 2011). Furthermore, many DEPs associated with inflammatory responses and glial cell activation have been identified. In addition, persistent chronic immune-inflammatory stimulation in PHN patients could further activate glial cells and aggravate nerve damage (Komori et al., 2007). Consistent with the metabolomics results, we observed significant upregulation of FAH and FAH-related biological pathways, including the metabolism of histidine, phenylalanine, tryptophan, and other amino acids (Weiss et al., 2018). Furthermore, consistent with the clinical changes, the cell structure- and integrity-related DEPs (such as the KRT family) in the PHN group were significantly reduced compared with the high keratin content in the HZ group, consistent with the disease process of skin healing in the PHN phase. The changes in metabolic substances in PHN patients revealed that chronic neuroimmune inflammation resulted in abnormal fatty and amino acid metabolism (glutamate-aspartate, tryptophan-kynurenine, and arginine-ornithine metabolic pathways), leading to the massive release of excitatory neurotransmitters and increased neurosensitivity involved in the development of PHN. Alterations in proteomics indicated that viral infection and inflammatory response persisted, leading to abnormal neurological function and glial cell activation.

In this study, we explored the metabolomic and proteomic signatures that transition from HZ to PHN. The expression of lipid metabolites involved in the inflammatory response was lower in patients with PHN. In contrast, the expression of amino acid metabolites involved in neurotransmitter anabolism was significantly higher than that in HZ patients. In addition, the metabolite pattern of profile 5 was the most crucial metabolite involved in lipid metabolism, exhibiting upregulation in HZ patients and downregulation in PHN patients. The expression patterns were consistent with that in the acute phase of neuroinflammation in the HZ state, and the body stabilizes neuronal cell membranes. It promotes neuronal repair by regulating glycerol phospholipid metabolism, whereas the downregulation of expression in the PHN state presented a chronic inflammatory phase and led to abnormal neural repair processes. In terms of proteomics, the DSP, JUP, and KRT families associated with skin alterations due to herpes were identified and reflected the healing process of lesions from the acute to the post-acute phase. This implied that the immune-inflammatory response owing to viral infection from HZ to PHN was one of the main pathological changes. More importantly, HSD17B10 is involved in branched-chain amino acid metabolism, SLC3A2 mediates the uptake of amino acids, and the downregulated ARG1 involved in arginine metabolism was identified as an important protein related to the synthesis and release of neurotransmitters and neuromodulators. This implies that the synthesis and release of neuromodulators and neurotransmitters could contribute to hyperalgesia, which might further explain the progression of HZ to PHN.

To better understand the clinical utility of differentiating metabolic patterns in HZ/PHN, we examined the correlation between the differential metabolites and clinical data. In patients with PHN, some lipid metabolites (PC and TG) were negatively correlated with pain scores, suggesting that the regulation of lipid metabolism in the immune-inflammatory response might be related to pain. In contrast, L-kynurenine in patients with PHN was positively correlated with disease duration, suggesting that the increased expression of L-kynurenine with prolongation of the disease might promote a significant release of neurotransmitters by aggravating the neuroinflammatory response and presenting the disease into the posterior phase. Interestingly, the increased expression of amino acid metabolites was more pronounced in women, while increased lipid metabolites were observed in men. This implies that amino acids and lipid metabolites are essential components in regulating pain in sex dimorphism.

Notably, we developed three optimal predictive models based on specific metabolic patterns and machine learning in classifying from healthy to HZ and PHN. The AUC of all these models were > 0.9. Accordingly, patients predicted to be at high risk of HZ/PHN, especially for HZ patients to develop PHN, may be considered for early prevention and clinical intervention. Moreover, the uniform standard could provide a more objective and measurable method than traditional methods based on age and accompanying disease (Wang X. X. et al., 2020). In a previous study, CE that ranked top in model 1 was associated with chronic postoperative pain (Lunde et al., 2020). Notably, N, N-dimethylarginine was identified in models 2 and 3. Furthermore, restoring arginine bioavailability through exogenous arginine supplementation might be a beneficial approach for treating PHN (Bakshi and Morris, 2016). Notably, a recent study proposed a novel classification method for patients with HZ and PHN based on functional magnetic resonance imaging (fMRI), which indicated that decreased brain activity *via* the SVM algorithm could be used to classify individuals with different pain conditions (Huang et al., 2020). Notably, ML models also provide a novel identification method for patients with herpetic neuralgia who are at risk of inadequate pain management. Studies have explored the clinical characteristics of herpetic neuralgia patients with medication responsiveness and built an optimal ML model to discriminate medication-resistant pain (MRP) from medication-sensitive pain (MSP) (Zhou et al., 2021). However, this study has some limitations. First, our multi-omics studies were only performed on plasma samples, owing to the impossibility of obtaining human peripheral or central nervous system samples in living individuals or difficulty obtaining sensitive skin tissue samples. Because the tissues and blood exhibited some inter-individual variations and correlation, plasma omics could only partly demonstrate the landscape of disease progression. Second, we did not perform validation tests with an expanded sample size for target proteins and metabolite identification *via* target metabolites, which will be our next research intention.

## Conclusion

Our study describes an integrative proteomics and metabolomics investigation of disease progression in patients with HZ and PHN. We proposed an optimal machine learning predictive model based on the clinical utility of metabolic patterns. Owing to the limited number of patients, future validation studies are needed to verify the findings reported in this study.

## Data availability statement

The original contributions presented in this study are publicly available. This data can be found here: http://www.proteomexchange.org/, PXD035009 and https://www.ebi.ac.uk/metabolights/, MTBLS5213.

## Ethics statement

The studies involving human participants were reviewed and approved by the Ethics Committee of West China Hospital of Sichuan university. The patients/participants provided their written informed consent to participate in this study.

## Author contributions

RHZ, JL, YJZ, HX, YXZ, and LY conceived and designed the study, wrote, and revised the manuscript. RHZ, JL, YJZ, and HX performed the experiments. RHZ, JL, and YJZ analyzed the data. All authors read the journal’s agreement and reviewed and approved the manuscript.
