# Supplementary material for: Characterization of plasma metabolites and proteins in patients with herpetic neuralgia and development of machine learning predictive models based on metabolomic profiling
Source: Front Mol Neurosci. 2022 Oct 6;15:1009677. doi: 10.3389/fnmol.2022.1009677 (PMC9583257; doi:10.3389/fnmol.2022.1009677)
Supplement: Supplementary file 1 [file Data_Sheet_1.docx]

| Groups | Pathway | Candidate metabolites with pathway annotation | All metabolites with pathway annotation | P value | Pathway ID | |
| --- | --- | --- | --- | --- | --- | --- |
| A-vs-B | Glycerophospholipid metabolism | 5 (23.81%) | 28 (4.93%) | 0.002369 | ko00564 |  |
|  | Retrograde endocannabinoid signaling | 3 (14.29%) | 14 (2.46%) | 0.012218 | ko04723 |  |
|  | Arachidonic acid metabolism | 2 (9.52%) | 8 (1.41%) | 0.031896 | ko00590 |  |
|  | Linoleic acid metabolism | 2 (9.52%) | 9 (1.58%) | 0.040100 | ko00591 |  |
|  | alpha-Linolenic acid metabolism | 2 (9.52%) | 10 (1.76%) | 0.049016 | ko00592 |  |
|  |  |  |  |  |  |  |
| A-vs-C | Caffeine metabolism | 3 (10%) | 14 (2.46%) | 0.032637 | ko00232 |  |
|  |  |  |  |  |  |  |
| B-vs-C | Oxidative phosphorylation | 2 (7.69%) | 5 (0.88%) | 0.018523 | ko00190 |  |
|  | Retrograde endocannabinoid signaling | 3 (11.54%) | 14 (2.46%) | 0.022179 | ko04723 |  |
|  | Glycerophospholipid metabolism | 4 (15.38%) | 28 (4.93%) | 0.033328 | ko00564 |  |
|  | Arachidonic acid metabolism | 2 (7.69%) | 8 (1.41%) | 0.047629 | ko00590 |  |

**Supplementary Table 1. Differential Metabolite KEGG Pathway Enrichment**

**Supplementary Table 2. Metabolites expression and function of profile 5**

| ID | Metabolites value Group A | Metabolites value Group B | Metabolites value Group C | Biological function | Name |
| --- | --- | --- | --- | --- | --- |
| NEG00005 | 0.9892 | 2.7243 | 0.9666 | Tricarboxylic acid cycle | Citric acid |
| POS00230 | 0.9936 | 2.2268 | 1.0720 | Lipid metabolism | N-Methylethanolamine phosphate |
| NEG04606 | 0.9872 | 2.0116 | 1.0407 | Lipid metabolism | Phosphatidylcholine (PC) |
| POS01678 | 1.0132 | 4.4798 | 0.7771 | Amino acid metabolism | N-Formimino-L-aspartate |
| NEG04583 | 1.0309 | 9.0114 | 0.9573 | Lipid metabolism | Phosphatidylethanolamine (PE) |
| NEG04569 | 1.1301 | 73.8528 | 0.9955 | Lipid metabolism | Glycerol diesters (DG) |
| POS04973 | 1.0143 | 2.9251 | 0.6453 | Lipid metabolism | Ceramide (Cer) |

Notes. Metabolites values indicating a fold change in expression versus control (Group A).

**Supplementary Table 3. Proteins expression and function of profile 4**

| ID | Proteins value Group A | Proteins value Group B | Proteins value Group C | Biological function | Symbol |
| --- | --- | --- | --- | --- | --- |
| ENST00000341511 | 0.968 | 0.972667 | 1.45 | Membrane signal transduction | ABCA2 |
| ENST00000457156 | 0.997333 | 1.07233 | 1.42833 | viral infection | HNRNPK |
| ENST00000621356 | 0.928667 | 0.944667 | 1.37467 | viral infection | RPS10 |
| ENST00000306390 | 0.931333 | 0.983333 | 1.359 | Neuroimmune inflammation | LRG1 |
| ENST00000616721 | 1.04467 | 1.06833 | 1.323 | Neuroimmune inflammation | FCGBP |
| ENST00000592860 | 1.06067 | 1.07533 | 1.321 | Complement system | CFD |
| ENST00000535296 | 0.993333 | 0.812 | 1.29 | Neuroimmune inflammation | SLC3A2 |
| ENST00000375298 | 0.940333 | 0.792 | 1.27733 | Branched chain amino acid metabolism | HSD17B10 |
| ENST00000618404 | 0.964333 | 0.891 | 1.261 | Chemokine | CCL14 |
| ENST00000435363 | 0.973 | 0.931333 | 1.24567 | Complement system | C4B |
| ENST00000611961 | 1.016 | 1.00233 | 1.233 | Platelet activation | GP1BA |
| ENST00000262776 | 0.963667 | 0.849 | 1.161 | immune reaction | LGALS3BP |
| ENST00000233072 | 0.823333 | 0.731333 | 1.14133 | Arginine metabolism | CPS1 |
| ENST00000569545 | 0.93 | 0.865667 | 1.07733 | Glucose metabolism | ALDOA |

Notes. Proteins values indicating a fold change in expression versus control (Group A).

**Supplementary Table 4. Proteins expression and function of profile 7**

| Gene ID | Proteins value Group A | Proteins value Group B | Proteins value Group C | Biological function | Symbol |
| --- | --- | --- | --- | --- | --- |
| ENST00000407106 | 1.05867 | 1.239 | 1.59133 | Amino acid metabolism | FAH |
| ENST00000233809 | 0.969667 | 1.13267 | 1.44633 | Neuroimmune inflammation | IGFBP2 |
| ENST00000376925 | 1.04733 | 1.13533 | 1.36733 | Neuroimmune inflammation | CST3 |
| ENST00000396620 | 1.04967 | 1.21233 | 1.36533 | Neuroimmune inflammation | CD163 |
| ENST00000376911 | 0.942667 | 1.18267 | 1.362 | Inflammatory chemotaxis | ANXA1 |
| ENST00000356495 | 0.982 | 1.126 | 1.345 | immune reaction | PIGR |
| ENST00000641975 | 0.987667 | 1.15533 | 1.318 | immune reaction | FCGBP |
| ENST00000313164 | 1.016 | 1.09067 | 1.25867 | Complement system | C7 |
| ENST00000393078 | 1.02233 | 1.11733 | 1.251 | Neuroimmune inflammation | SERPINA3 |

Notes. Proteins values indicating a fold change in expression versus control (Group A).

**Supplementary Table 5. Loading values of differential expressed metabolites and proteins in Group A VS B**

| Classification | Name | Loading_1 | Loading_2 | Square sum |
| --- | --- | --- | --- | --- |
| Metabolites | 3-methyl pyruvic acid | -0.061690794 | -0.188797652 | 0.039450307 |
|  | Histidine | 0.028781068 | 0.157171215 | 0.025531141 |
|  | sn-Glycero-3-phosphocholine | 0.068217448 | 0.113130113 | 0.017452043 |
|  | PS(14:0/20:4) | -0.043845039 | 0.048516848 | 0.004276272 |
|  | PC(14:0/18:1(11Z)) | -0.045450974 | 0.042892638 | 0.003905569 |
|  | CerP(d18:1/20:0) | -0.047250515 | 0.035684477 | 0.003505993 |
|  | PE(14:0/18:1(11Z)) | -0.047739643 | 0.034764006 | 0.00348761 |
|  | N-Formimino-L-aspartate | -0.050164906 | 0.02882658 | 0.003347489 |
|  | DG(18:2n6/0:0/22:6n3) | -0.05143551 | 0.024827207 | 0.003262002 |
|  | Citric acid | -0.055928634 | 0.006706304 | 0.003172987 |
|  | Itaconic acid | -0.047699299 | 0.015595421 | 0.00251844 |
|  |  |  |  |  |
| Proteins | S100A9 | -0.514248381 | 0.207424985 | 0.307476521 |
|  | MYH1 | -0.44304694 | 0.053865975 | 0.199192135 |
|  | FBLN1 | -0.438765831 | 0.067840148 | 0.19711774 |
|  | GP5 | 0.374438729 | -0.045227477 | 0.142249886 |
|  | PPBP | 0.326488949 | -0.026090238 | 0.107275734 |
|  | JUP | -0.011139859 | -0.259658017 | 0.067546382 |
|  | ALDOB | 0.020544411 | -0.24801721 | 0.061934609 |
|  | KRT5 | -0.058479196 | -0.237744084 | 0.059942066 |
|  | DSG1 | -0.206980778 | -0.129614091 | 0.059640855 |
|  | KRT2 | -0.039878997 | -0.236571156 | 0.057556246 |
|  | KRT9 | -0.034559392 | -0.237355455 | 0.057531964 |
|  | HRNR | -0.073840596 | -0.22716182 | 0.057054926 |
|  | DSP | -0.111976131 | -0.203709281 | 0.054036125 |
|  | KRT10 | -0.096104606 | -0.210563154 | 0.053572937 |
|  | FLG2 | -0.059861441 | -0.182449557 | 0.036871233 |

Notes. Loading values indicated the explanatory power of the variable (metabolite/protein) in each group (i.e., the degree of contribution to the difference between groups), and positive or negative loading values indicated positive or negative association with another group study.

**Supplementary Table 6. Loading values of differential expressed metabolites and proteins in Group A VS C**

| Classification | Name | Loading_1 | Loading_2 | Square sum |
| --- | --- | --- | --- | --- |
| Metabolites | 5,6-dehydro Arachidonic Acid | 0.0345281 | 0.121697798 | 0.016002548 |
|  | D-Gluconic acid | -0.101108 | -0.051951299 | 0.012921894 |
|  | sn-Glycero-3-phosphocholine | 0.0724958 | 0.075647823 | 0.010978238 |
|  | D-Ornithine | -0.099206 | -0.02657245 | 0.010547994 |
|  | Homoarginine | 0.0643050 | 0.077864148 | 0.010197959 |
|  | Pyroglutamic acid | -0.067550 | 0.069813975 | 0.009437077 |
|  | Linoelaidic Acid | 0.0952988 | 0.018485298 | 0.009423579 |
|  | Hydroxyphenyllactic acid | -0.060920 | 0.074594985 | 0.009275678 |
|  | L-KYNURENINE | -0.089084 | 0.028899122 | 0.008771177 |
|  | Ornithine | -0.087637 | 0.017180566 | 0.007975484 |
|  | 5-Hydroxytryptophol glucuronide | 0.0780893 | 0.040569045 | 0.007743801 |
|  | Phenylacetylglutamine | -0.043590 | -0.071876105 | 0.007066319 |
|  | Oleoyl Ethanolamide | -0.016829 | 0.081544766 | 0.006932767 |
|  | Oxaloacetate | 0.0387777 | -0.066957422 | 0.005987007 |
|  | 2-Oxoglutaramate | -0.064549 | 0.03442556 | 0.005351717 |
|  | Selenohomocysteine | -0.039049 | -0.02742008 | 0.002276726 |
|  |  |  |  |  |
| Proteins | LRG1 | -0.128215562 | 0.294363707 | 0.103089222 |
|  | S100A8 | -0.136365892 | 0.235193603 | 0.073911687 |
|  | FAH | -0.097103908 | -0.235182396 | 0.064739929 |
|  | SERPINA3 | -0.130743557 | 0.211135727 | 0.061672173 |
|  | C7 | -0.126524349 | 0.177003411 | 0.047338618 |
|  | HNRNPK | -0.113703185 | -0.164511836 | 0.039992558 |
|  | ANXA1 | -0.094003881 | -0.170442782 | 0.037887472 |
|  | FBLN1 | -0.102715711 | -0.158699797 | 0.035736143 |
|  | FCGBP | -0.139810444 | 0.116823241 | 0.03319463 |
|  | KNG1 | 0.137871946 | -0.103294147 | 0.029678354 |
|  | RPS10 | -0.119299487 | -0.123422734 | 0.029465539 |
|  | PIGR | -0.134212165 | 0.091294179 | 0.026347533 |
|  | CD163 | -0.133960528 | 0.044157073 | 0.01989527 |
|  | ABCA2 | -0.13647579 | 0.030904359 | 0.019580721 |
|  | SELENOP | 0.134872495 | -0.036449591 | 0.019519162 |
|  | CFD | -0.126917925 | 0.0565707 | 0.019308404 |
|  | CST3 | -0.133284412 | 0.033804813 | 0.0189075 |
|  | KRT9 | 0.13090532 | 0.041158591 | 0.018830233 |
|  | IGFBP2 | -0.123897623 | -0.0582256 | 0.018740841 |
|  | GP1BA | -0.133168229 | 0.017775694 | 0.018049753 |
|  | KRT2 | 0.126576715 | 0.036440238 | 0.017349556 |
|  | C4B | -0.117801773 | 0.006329514 | 0.01391732 |

Notes. Loading values indicated the explanatory power of the variable (metabolite/protein) in each group (i.e., the degree of contribution to the difference between groups), and positive or negative loading values indicated positive or negative association with another group study.

**Supplementary Table 7. Loading values of differential expressed metabolites and proteins in Group B VS C**

| Classification | Name | Loading_1 | Loading_2 | Square sum |
| --- | --- | --- | --- | --- |
| Metabolites | Histidine | -0.052203038 | 0.142586781 | 0.023056147 |
|  | Homoarginine | 0.069376898 | -0.126578072 | 0.020835162 |
|  | Selenohomocysteine | -0.090246931 | 0.109337165 | 0.020099124 |
|  | Oleoyl Ethanolamide | -0.067595892 | 0.094599955 | 0.013518356 |
|  | 2-Oxoglutaramate | -0.041473614 | 0.070266375 | 0.006657424 |
|  | PS(14:0/20:4) | 0.036537334 | 0.054149881 | 0.004267186 |
|  | PC(14:0/18:1(11Z)) | 0.036795071 | 0.050231986 | 0.00387713 |
|  | CerP(d18:1/20:0) | 0.033993433 | 0.048602416 | 0.003517748 |
|  | PE(14:0/18:1(11Z)) | 0.044039321 | 0.034011727 | 0.003096259 |
|  | Citric acid | 0.043343867 | 0.033106458 | 0.002974728 |
|  | N-Formimino-L-aspartate | 0.05313965 | 0.010983048 | 0.00294445 |
|  | lysoPC(26:1(5Z)) | -0.047650455 | -0.017336264 | 0.002571112 |
| Proteins | Itaconic acid | 0.039164357 | 0.028633025 | 0.002353697 |
|  |  |  |  |  |
|  | ARG1 | 0.028289464 | -0.218848686 | 0.048695041 |
|  | PF4 | -0.150706999 | -0.111244119 | 0.035087854 |
|  | DSP | 0.053436188 | -0.175563943 | 0.033678124 |
|  | GP1BA | -0.024753782 | 0.177323887 | 0.03205651 |
|  | SELENOP | 0.041716267 | -0.163230453 | 0.028384428 |
|  | C4B | -0.141172234 | -0.076941543 | 0.025849601 |
|  | ALDOA | -0.139918383 | -0.065226185 | 0.023831609 |
|  | KNG1 | 0.133139463 | 0.069073666 | 0.022497288 |
|  | GP5 | -0.139644265 | -0.039168156 | 0.021034665 |
|  | FLG2 | 0.047577603 | -0.134139695 | 0.020257086 |
|  | CPS1 | -0.135688707 | -0.042106857 | 0.020184413 |
|  | PPBP | -0.136003887 | -0.033053 | 0.019589558 |
|  | DSG1 | 0.090536086 | -0.102936176 | 0.018792639 |
|  | CFD | -0.12969421 | 0.001490539 | 0.01682281 |
|  | CST3 | -0.092282232 | 0.088374749 | 0.016326107 |
|  | FCGBP | -0.083574714 | 0.09108421 | 0.015281066 |
|  | CCL14 | -0.109636496 | -0.040782232 | 0.013683352 |
|  | HNRNPK | -0.107006713 | -0.029914281 | 0.012345301 |
|  | LTBP1 | -0.11089772 | -0.00326031 | 0.012308934 |
|  | RPS10 | -0.091619565 | 0.021221233 | 0.008844485 |
|  | SLC3A2 | -0.090957046 | 0.021119623 | 0.008719223 |
|  | LGALS3BP | -0.090121769 | 0.013692605 | 0.008309421 |
|  | HSD17B10 | -0.085503042 | 0.028104464 | 0.008100631 |

Notes. Loading values indicated the explanatory power of the variable (metabolite/protein) in each group (i.e., the degree of contribution to the difference between groups), and positive or negative loading values indicated positive or negative association with another group study.
