# Supplementary material for: Characterization of plasma metabolites and proteins in patients with herpetic neuralgia and development of machine learning predictive models based on metabolomic profiling
Source: Front Mol Neurosci. 2022 Oct 6;15:1009677. doi: 10.3389/fnmol.2022.1009677 (PMC9583257; doi:10.3389/fnmol.2022.1009677)
Supplement: Supplementary file 2 [file Data_Sheet_2.docx]

Supplementary Material

# Supplementary Figures and Tables

## Supplementary Figures

**Supplementary Figure 1. Base peak chromatogram of QC and blank samples**

A–B) Base peak chromatogram diagram of the QC sample in NEG and POS modes; C–D) Base peak chromatogram diagram of blank samples in NEG and POS modes. The abscissa represents the time, and the ordinate represents the relative abundance of metabolites.

**Supplementary Figure 2. PCA score plots of NEG and POS modes among the groups**

A–C) PCA score plot in group A vs. B, group A vs. C, and group B vs. C of NEG mode, respectively; D–F) PCA score plot in group A vs. B, group A vs. C, and group B vs. C of POS mode, respectively.

**Supplementary Figure 3. PLS-DA score plots of NEG and POS modes between groups**

A–C) PLS-DA score plot in group A vs. B, group A vs. C, and group B VS C of NEG mode, respectively; D–F) PLS-DA score plot in group A vs. B, group A vs. C, and group B vs. C of POS mode, respectively.

**Supplementary Figure 4. Permutation test plots of NEG and POS modes between the groups**

A–C) Permutation test plot in group A vs. B, group A vs. C, and group B vs. C of NEG mode, respectively; D–F) Permutation test plot in group A vs. B, group A vs. C, and group B vs. C of POS mode, respectively.

**Supplementary Figure 5. KEGG and GO enrichment analysis of differentially expressed proteins between groups**

A) KEGG and B) Gene ontology (GO) term (including biological process, cellular component, and molecular function) enrichment for differentially expressed proteins (DEPs) in group A vs. B. C) KEGG and D) GO term enrichment for DEPs in group A vs. C. E) KEGG and F) GO term enrichment for DEPs in group B vs. C.

## Supplementary Tables

**Supplementary Table 1. Differential Metabolite KEGG Pathway Enrichment**

**Supplementary Table 2. Metabolites expression and function of profile 5**

**Supplementary Table 3. Proteins expression and function of profile 4**

**Supplementary Table 4. Proteins expression and function of profile 7**

**Supplementary Table 5. Loading values of differential expressed metabolites and proteins in Group A vs. B**

**Supplementary Table 6. Loading values of differential expressed metabolites and proteins in Group A vs. C**

**Supplementary Table 7. Loading values of differential expressed metabolites and proteins in Group B vs. C**

**1.3 Supplemental and methods**

**1.3.1 Untargeted metabolomics analysis**

**1) Metabolite extraction and detection:**

50 mg of sample was weighted to an EP tube. After the addition of 1000 μL of extract solvent (acetonitrile-methanol-water, 2:2:1, containing internal standard), the samples were vortexed for 30 s, homogenized at 45 Hz for 4 min, and sonicated for 5 min in ice-water bath. The homogenate and sonicate circle were repeated for 3 times, followed by incubation at -20 ℃ for 1 h and centrifugation at 12000 rpm and 4 ℃ for 15 min. The resulting supernatants were transferred to Liquid chromatography-mass spectrometry (LC-MS) vials. The quality control (QC) sample was prepared by mixing an equal aliquot of the supernatants from all of the samples.

LC-MS/MS analyses were performed using an UHPLC system (1290, Agilent Technologies) with a UPLC HSS T3 column (2.1 mm × 100 mm, 1.8 μm) coupled to Q Exactive (Orbitrap MS, Thermo). The mobile phase A was 0.1% formic acid in water for positive, and 5 mmol/L ammonium acetate in water for negative, and the mobile phase B was acetonitrile. The elution gradient was set as follows: 0 min, 1% B; 1 min, 1% B; 8 min, 99% B; 10 min, 99% B; 10.1 min, 1% B; 12 min, 1% B. The flow rate was 0.5 mL/min. The injection volume was 2 μL. The QE mass spectrometer was used for its ability to acquire MS/MS spectra on an information-dependent basis (IDA) during an LC/MS experiment. In this mode, the acquisition software (Xcalibur 4.0.27, Thermo) continuously evaluates the full scan survey MS data as it collects and triggers the acquisition of MS/MS spectra depending on preselected criteria. ESI source conditions were set as following: Sheath gas flow rate as 45 Arb, Aux gas flow rate as 15Arb, Capillary temperature 320 ℃, Full ms resolution as 70000, MS/MS resolution as 17500, Collision energy as 20/40/60 eV in NCE model, Spray Voltage as 3.8 kV (positive) or -3.1 kV (negative), respectively.

**2) Data preprocessing and annotation**

MS raw data(.raw) files were converted to the mzML format using ProteoWizard, and processed by R package XCMS (version 3.2)[1], including retention time alignment, peak detection and peak matching. Then the data were filtered by the followed criterion: sample numbers contains a metabolite was less than 50% all sample numbers in a group (QC were also taken as a group). Normalization to an internal standard[2] for each sample was done subsequently. Next, missing values were replaced by the half of the minimum value found in the dataset by default[3]. The preprocessing results generated a data matrix that consisted of the retention time (RT), mass-to-charge ratio (m/z) values, and peak intensity. OSI-SMMS (version 1.0, Dalian Chem Data Solution Information Technology Co. Ltd.) was used for peak annotation after data processing with in-house MS/MS database.

**3) Multivariate statistical analysis**

**PCA:** For a preliminary visualization of differences between different groups of samples, the unsupervised dimensionality reduction method principal component analysis (PCA) was applied in all samples using R package models (http://www.r-project.org/). PCA is a statistical procedure that converts hundreds of thousands of correlated metabolites variables into a set of values of linearly uncorrelated variables called principal components.

**PLS-DA****:** Partial least squares discriminant analysis (PLS-DA) is a supervised dimensionality reduction method in which class memberships are coded in matrix form into Y to better distinguish the metabolomics profile of two groups by screening variables correlated to class memberships[4]. PLS-DA was applied in comparison groups using R package models (<http://www.r-project.org/>).

**OPLS-DA****:** Orthogonal projection to latent structures-discriminant analysis (OPLS-DA). OPLS-DA is an extension of PLS-DA which incorporates an Orthogonal Signal Correction (OSC) filter into a PLS model. The basic concept in OPLS is to separate the systematic variation in X into two parts, one that is correlated to Y and one that is not correlated (orthogonal) with Y. Only the Y‐predictive variation is used to model the data. OPLS-DA was applied in comparison groups using R package models (http://www.r-project.org/). The OPLS-DA model was further validated by cross-validation and 200 permutation test [5]. For cross-validation, the data was partitioned into seven subsets, where each of the subsets was then used as a validation set. R2 indicated the total variation in the data matrix that was explained by the model. Predictive ability (Q2) values represented the most recognized diagnostic statistical parameter to validate the OPLS-DA model in metabolomics. Acceptable predictive model is considered for Q2 value greater than 0.4. Good predictive model is considered for Q2 value greater than 0.9. Permutation test randomly permutes class labels for 200 times and then produces a distribution of R2’ values and Q2’ values. In essence, a reliable model should yield significantly larger R2 and Q2 value compared to R2’ and Q2’ values generated from random models using the same data set.

**1.3.2 iTRAQ Protein Quantification Method**

**1) Protein extraction and quantification:**

Samples were transferred into lysis buffer (2% SDS, 7M urea, 1mg/ml protease inhibitor cocktail), and homogenized for 3min (180 seconds three times) in ice using a ultrasonic homogenizer. The homogenate was centrifuged at 15000rpm for 15min at 4℃, and the supernatant was collected. The quantitative accuracy and quantify protein extraction were evaluated by SDS PAGE electrophoresis.

**2) Protein digestion and iTRAQ labelling**

BCA Protein Assay Kit was used to determine the protein concentration of the supernatant. 100 μg protein per condition was transferred into a new tube and adjusted to a final volume of 100μL with 8M Urea. 11μL of 1M DTT (DL-Dithiothreitol) was added and samples were incubated at 37℃ for 1 hour. Then samples were centrifugated at 14000 rpm for 10min in a 10K ultrafiltration tube (Millipore). 120μL of the 55mM iodoacetamide was added to the sample and incubated for 20 minutes protected from light at room temperature.

For each sample, 8M urea were replaced by 10mM TEAB (triethylammonium bicarbonate) by centrifugating three times. Proteins were then tryptic digested with sequence-grade modified trypsin (Promega, Madison, WI) at 37°C overnight. Then the digested samples were centrifugated at 13500 rpm for 12min, dried in vacuum, and dissolved in 500mM TEAB. The resultant peptide mixture was labeled with iTRAQ/TMT tags (iTRAQ Reagents-8Plex(SCIEX)) for 2h at room temperature. The labeled samples were combined and dried in vacuum.

**3) High PH Reverse Phase Separation**

The peptide mixture was re-dissolved in buffer A (buffer A: 20mM ammonium formate in water, pH10.0, adjusted with ammonium hydroxide), and then fractionated by high pH separation using Ultimate 3000 system (Thermo Fisher scientific, MA, USA) connected to a reverse phase column (XBridge C18 column, 4.6mm x 250 mm, 5μm, Waters Corporation, MA, USA). High pH separation was performed using a linear gradient starting from 5% B to 45% B in 40 min (buffer B: 20mM ammonium formate in 80% ACN, pH 10.0, adjusted with ammonium hydroxide). The column was re-equilibrated at initial conditions for 15 min. The column flow rate was maintained at 1mL/min and column temperature was maintained at 30℃. Twelve fractions were collected; each fraction was dried in a vacuum concentrator for the next step.

**4) Low PH nano-HPLC-MS/MS analysis (Orbitrap Fusion)**

Peptide fractions were resuspended with 30μl solvent C respectively (C: water with 0.1% formic acid; D: ACN with 0.1% formic acid), separated by nanoLC and analyzed by on-line electrospray tandem mass spectrometry. The experiments were performed on an Easy-nLC 1000 system (Thermo Fisher Scientific, MA, USA) connected to a Orbitrap Fusion Tribrid mass spectrometer (Thermo Fisher Scientific, MA, USA) equipped with an online nano-electrospray ion source. 10μl peptide sample was loaded onto the trap column (Thermo Scientific Acclaim PepMap C18, 100μm x 2cm), with a flow of 10μl/min for 3 min and subsequently separated on the analytical column (Acclaim PepMap C18, 75μm x 15cm) with a linear gradient, from 2% D to 40% D in 70 min. The column was re-equilibrated at initial conditions for 10 min. The column flow rate was maintained at 300nL/min. The electrospray voltage of 2kV versus the inlet of the mass spectrometer was used.

The fusion mass spectrometer was operated in the data-dependent acquisition mode to switch automatically between MS and MS/MS acquisition. Full-scan MS spectra (m/z 350-1550) were acquired with a mass resolution of 120K, followed by sequential high energy collisional dissociation (HCD) MS/MS scans with a resolution of 30K. The intense signals in the MS spectra (>1e4) underwent an additional MS/MS analysis. The automatic gain controls (AGC) for the MS and MS/MS were set to 4e5 and 8e4, respectively. The maximum ion injection times for the MS and MS/MS were 50 and 100 ms, respectively. The isolation window was set as 1.6 Da. In all cases, one microscan was recorded using dynamic exclusion of 30 seconds.

**5) Low PH nano-HPLC-MS/MS analysis (Q-Exactive)**

Peptide fractions were resuspended with 30μl solvent C respectively (C: water with 0.1% formic acid; D: ACN with 0.1% formic acid), separated by nanoLC and analyzed by on-line electrospray tandem mass spectrometry. The experiments were performed on an Easy-nLC 1000 system (Thermo Fisher Scientific, MA, USA) connected to a Q Exactive mass spectrometer (Thermo Fisher Scientific, MA, USA) equipped with an online nano-electrospray ion source. 10μl peptide sample was loaded onto the trap column (Thermo Scientific Acclaim PepMap C18, 100μm x 2cm), with a flow of 10μl/min for 3 min and subsequently separated on the analytical column (Acclaim PepMap C18, 75μm x 15cm) with a linear gradient, from 2% D to 40% D in 70 min. The column was re-equilibrated at initial conditions for 10 min. The column flow rate was maintained at 300nL/min. The electrospray voltage of 2kV versus the inlet of the mass spectrometer was used. The fusion mass spectrometer was operated in the data-dependent acquisition mode to switch automatically between MS and MS/MS acquisition. Full-scan MS spectra (m/z 350-1550) were acquired with a mass resolution of 35K, followed by sequential high energy collisional dissociation (HCD) MS/MS scans with a resolution of 17.5K. The intense signals in the MS spectra (>2e4) underwent an additional MS/MS analysis.

The automatic gain controls (AGC) for the MS and MS/MS were set to 3e6 and 1e5, respectively. The maximum ion injection times for the MS and MS/MS were 60 and 50ms, respectively. The isolation window was set as 1.6 Da. In all cases, one microscan was recorded using dynamic exclusion of 30 seconds.

**6) Database search**

Tandem mass spectra were extracted, charge state deconvoluted and deisotoped by Mascot Distiller version 2.6. Then the mass spectrometry data were transformed into MGF files with Proteome Discovery 1.2 (Thermo, Pittsburgh, PA, USA) and analyzed using Mascot search engine (Matrix Science, London, UK; version 2.3.2). Mascot database was set up for protein identification using human database in NCBInr/SwissProt/Uniprot/IPI assuming the digestion enzyme trypsin with 1 missed cleavage allowed. Mascot was searched with a fragment ion mass tolerance of 0.050 Da and a parent ion tolerance of 20.0 PPM. Carbamidomethyl of cysteine and iTRAQ8plex of lysine and the n-terminus were specified in Mascot as fixed modifications. Deamidated of asparagine and glutamine, oxidation of methionine and acetyl of the n-terminus were specified in Mascot as variable modifications.

**7) Protein identification and quantification**

Protein identifications were accepted if they could achieve an FDR less than 1.0% by the Scaffold Local FDR algorithm. Proteins that contained similar peptides and could not be differentiated based on MS/MS analysis alone were grouped to satisfy the principles of parsimony. Protein quantification was carried out in those proteins identified in all the samples with unique spectra ≥2. Protein relative quantification was based on the ratios of reporter ions, which reflect the relative abundance of peptides. The Mascot search results were averaged using medians and quantified. Proteins with fold change in a comparison ＞ 1.2 or ＜ 0.83 and unadjusted significance level p < 0.05 were considered differentially expressed.

**1.3.3 Integrative Proteomics-Metabolomics analysis**

**1) O2PLS model**

In order to integrate the proteomics and metabolomic data, we performed a Two-way Orthogonal PLS (O2PLS) analysis[6]. This method decomposes the variation present in the two data matrices into three parts, the joint variation between the two datasets, the orthogonal variation that is unique to each dataset and noise. The model assumes that some latent variables are responsible for the variation in the joint and orthogonal parts. O2PLS models were calculated using the OmicsPLS package of R[2]. To determine the optimal number of components, the proposed alternative cross-validation procedure was utilized[7]. The best models were used for integration analysis.

**2) Pearson model**

Pearson correlation coefficients were calculated for metabolome and proteomics data integration. Protein and metabolite pairs were ranked in the descending order of absolute correlation cofficients. The top 50 genes and metabolites were selected for heatmap analysis using pheatmap packages in R project.

1. Smith, C.A., et al., *XCMS: processing mass spectrometry data for metabolite profiling using nonlinear peak alignment, matching, and identification.* Anal Chem, 2006. **78**(3): p. 779-87.

2. Roberts, L.D., et al., *Targeted metabolomics.* Curr Protoc Mol Biol, 2012. **Chapter 30**: p. Unit 30.2.1-24.

3. Xia, J., et al., *MetaboAnalyst: a web server for metabolomic data analysis and interpretation.* Nucleic Acids Res, 2009. **37**(Web Server issue): p. W652-60.

4. Worley, B. and R. Powers, *Multivariate Analysis in Metabolomics.* Curr Metabolomics, 2013. **1**(1): p. 92-107.

5. Triba, M.N., et al., *PLS/OPLS models in metabolomics: the impact of permutation of dataset rows on the K-fold cross-validation quality parameters.* Mol Biosyst, 2015. **11**(1): p. 13-9.

6. Bylesjö, M., et al., *Data integration in plant biology: the O2PLS method for combined modeling of transcript and metabolite data.* Plant J, 2007. **52**(6): p. 1181-91.

7. Bouhaddani, S.E., et al., *Evaluation of O2PLS in Omics data integration.* BMC Bioinformatics, 2016. **17 Suppl 2**(Suppl 2): p. 11.
