# Supplementary figures and images for: Characterization of plasma metabolites and proteins in patients with herpetic neuralgia and development of machine learning predictive models based on metabolomic profiling
Source: Front Mol Neurosci. 2022 Oct 6;15:1009677. doi: 10.3389/fnmol.2022.1009677 (PMC9583257; doi:10.3389/fnmol.2022.1009677)

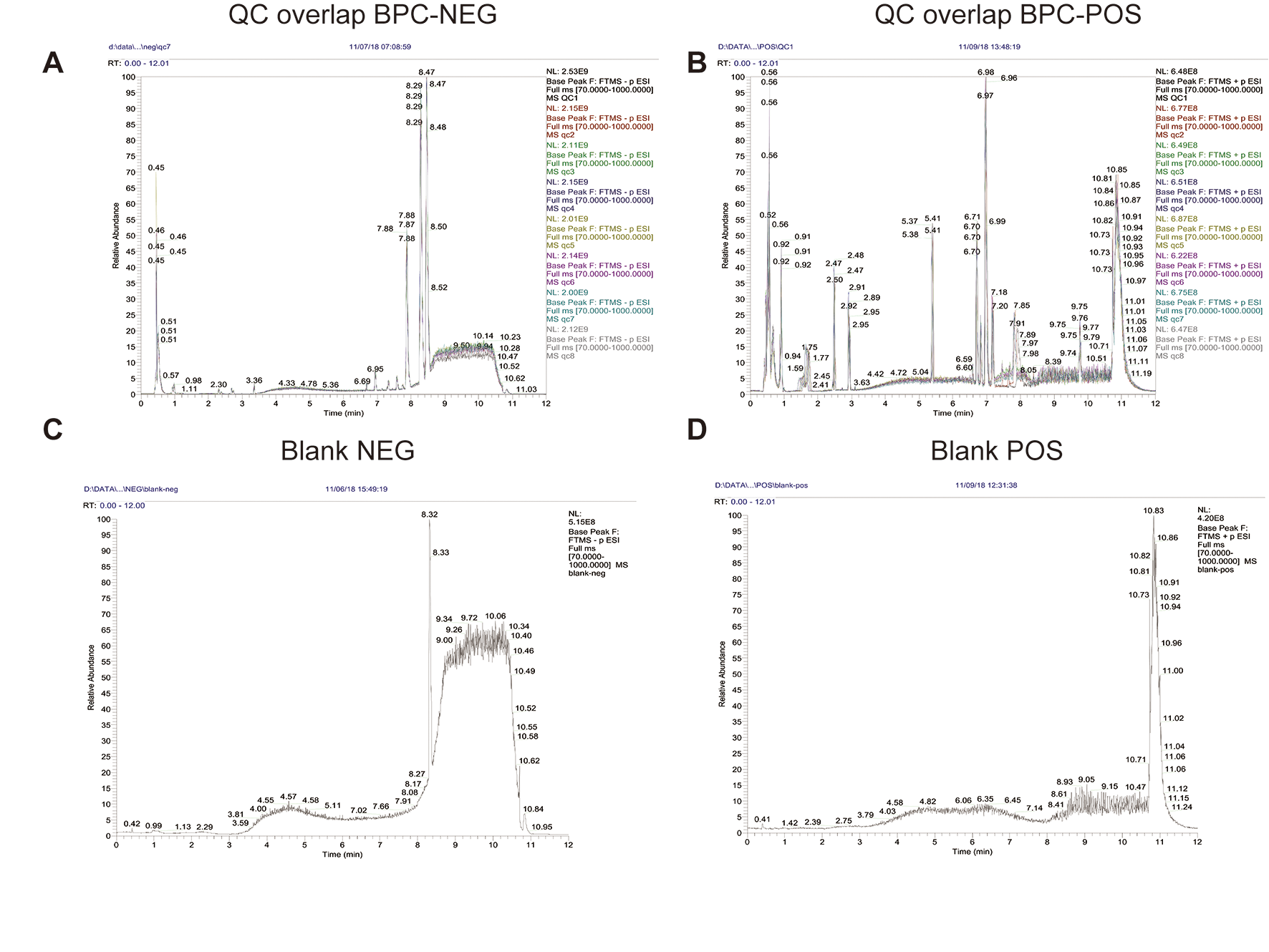

Supplement: Supplementary file 3 [file Image_1.TIF]

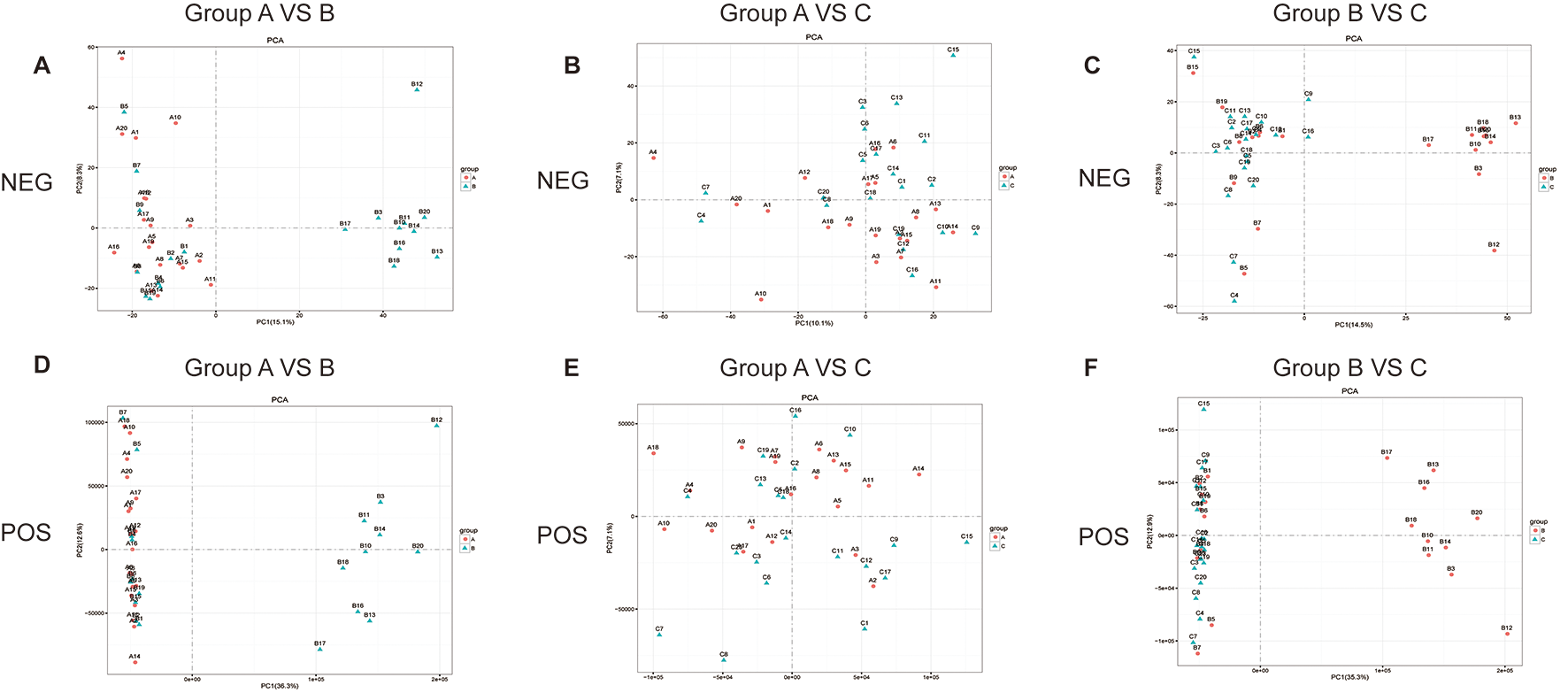

Supplement: Supplementary file 4 [file Image_2.TIF]

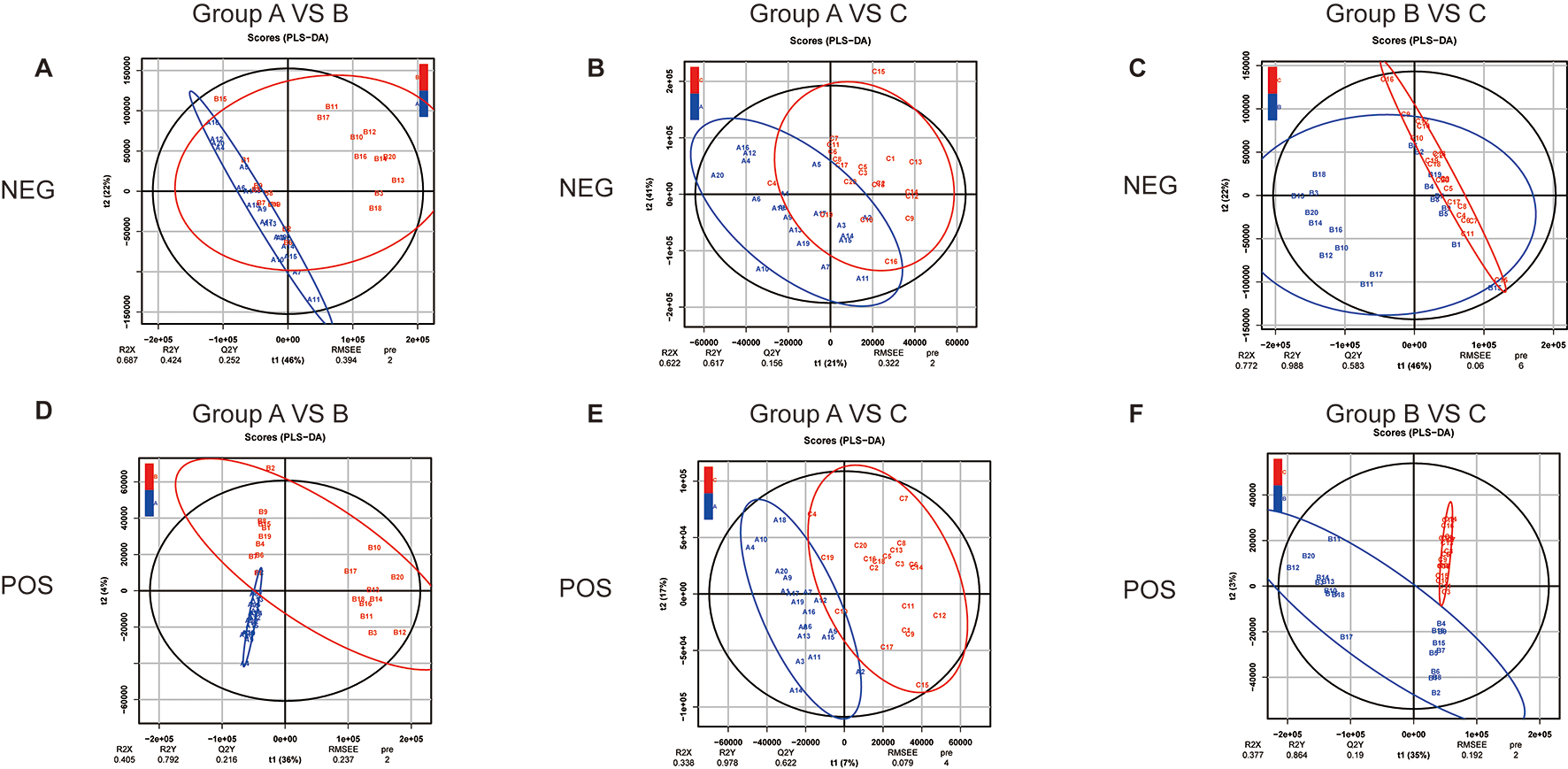

Supplement: Supplementary file 5 [file Image_3.TIF]

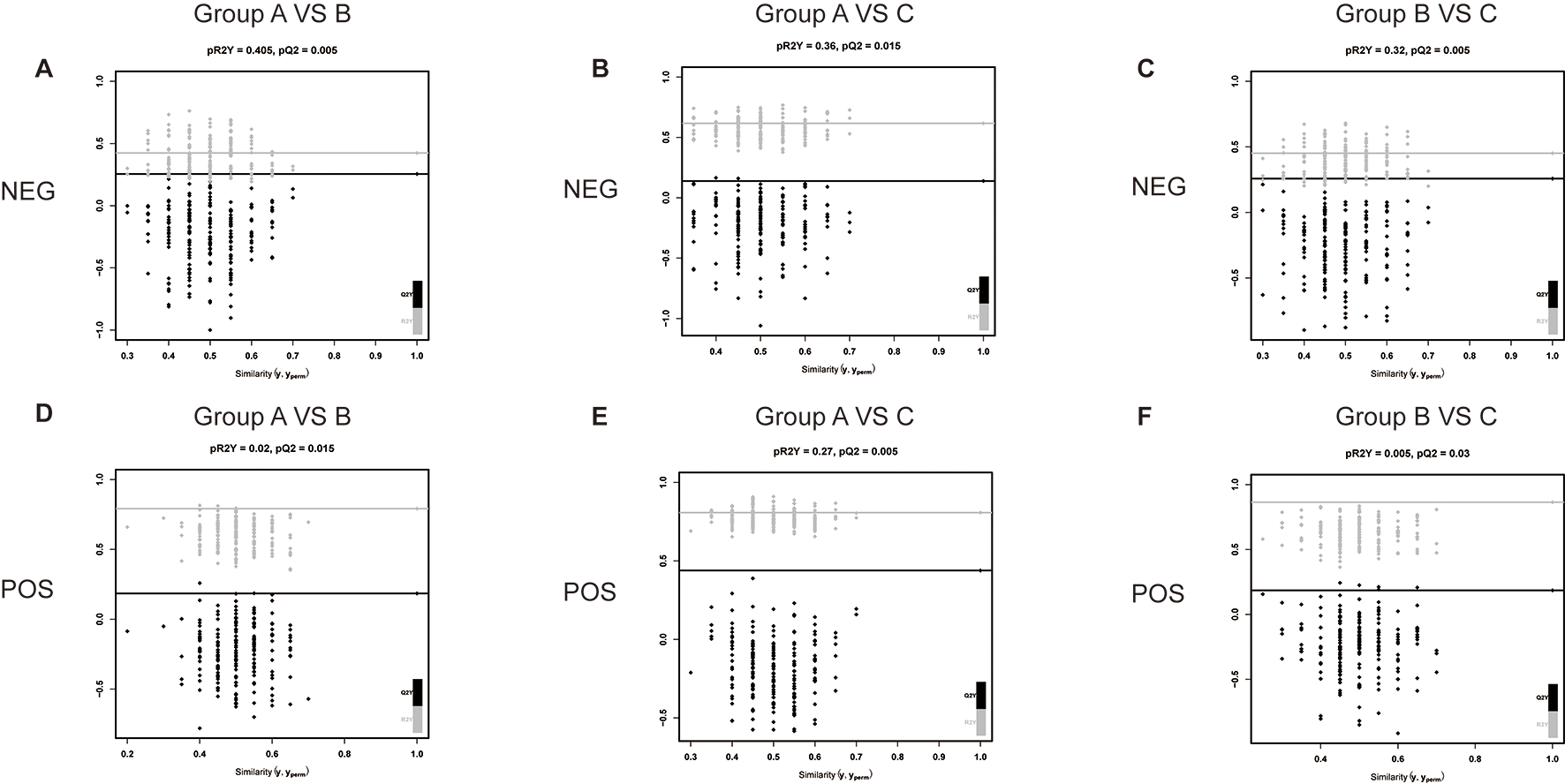

Supplement: Supplementary file 6 [file Image_4.TIF]

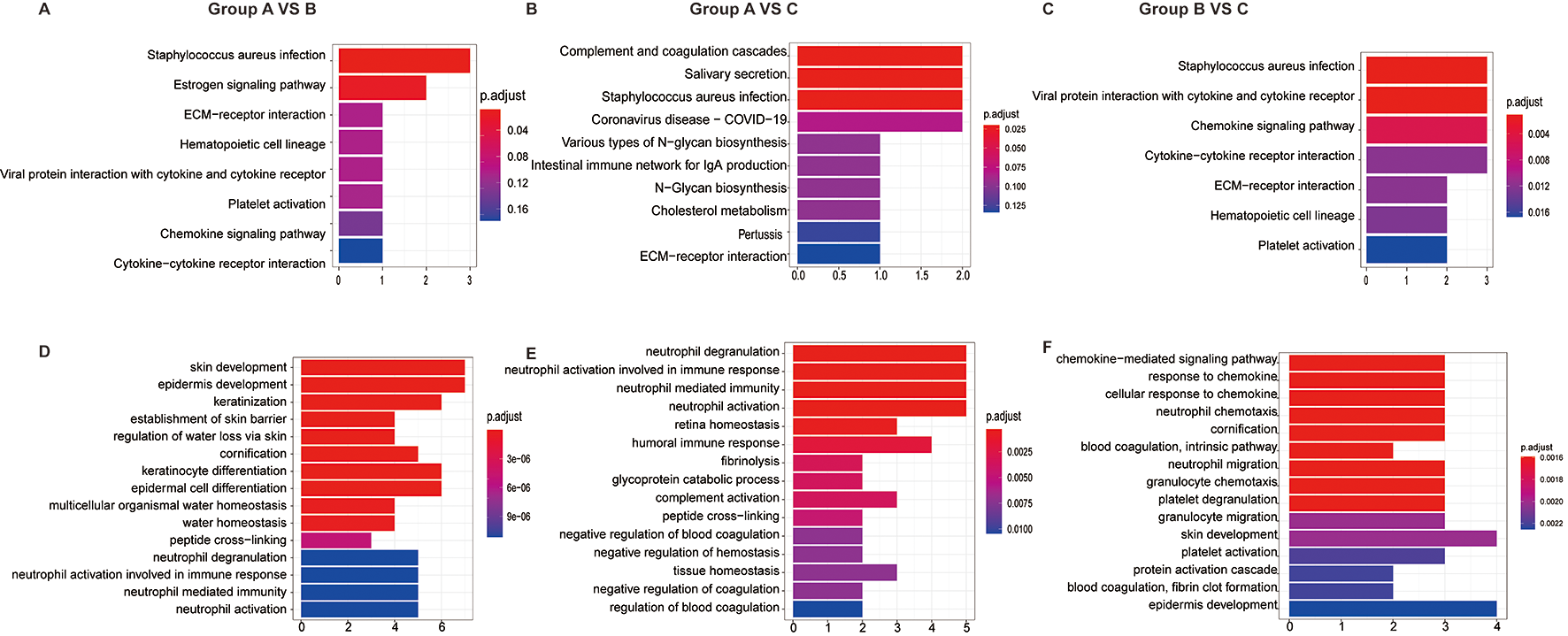

Supplement: Supplementary file 7 [file Image_5.TIF]
